# COMPUTERIZED COGNITIVE TRAINING, WITH OR WITHOUT EXERCISE, TO PROMOTE COGNITIVE FUNCTION: A RANDOMIZED TRIAL

**DOI:** 10.1093/geroni/igz038.215

**Published:** 2019-11-08

**Authors:** Lisanne F ten Brinke, John R Best, Joey L Chan, Cheyenne Ghag, Kirk I Erickson, Todd C Handy, Teresa Liu-Ambrose

**Affiliations:** 1 University of British Columbia, Vancouver, British Columbia, Canada; 2 University of Pittsburgh, Pittsburgh, Pennsylvania, United States

## Abstract

Given the world’s aging population, it is important to identify strategies that promote healthy cognitive aging. Computerized cognitive training (CCT) may be a promising method to combat cognitive decline in older adults. Moreover, physical exercise immediately prior to CCT might provide additional cognitive benefits. We conducted a randomized controlled trial to examine the effect of a CCT intervention, alone or preceded by physical exercise, on memory and executive functions in older adults. 124 community-dwelling older adults aged 65-85 years were randomly assigned to either 8-weeks of: 1) 3x/week group-based CCT plus 3x/week CCT sessions at home; 2) 3x/week group-based CCT combined with a 15-minute brisk walk (Ex-CCT) plus 3x/week Ex-CCT sessions at home; or 3)3x/week group-based sham exercise and education sessions (CON). At baseline and 8-weeks standard neuropsychological tests of verbal memory and learning and executive functions were administered, including the Rey Auditory Verbal Learning Test (RAVLT), Stroop test, Flanker test, Trail Making Tests (TMT B-A), and Dimensional Change Card Sort (DCCS) Test. At trial completion, there were no differences in RAVLT performance. Compared with CON, FBT and Ex-FBT participants significantly improved performance on the Stroop test (p = .001 and p = .023, respectively). Additionally, those randomized to Ex-CCT improved performance on the Flanker test (p = .002), TMT B-A (p = .047), and the DCCS Test (p = .023) compared with BAT. These findings suggest that an 8-week CCT program could benefit executive functions, and that implementing exercise immediately prior to CCT could provide broader benefits.

